# Bibliometric research on analysis of links between periodontitis and cardiovascular diseases

**DOI:** 10.3389/fcvm.2023.1255722

**Published:** 2023-09-04

**Authors:** Kuangyun Tang, Yongjia Wu, Qianhan Zheng, Xuepeng Chen

**Affiliations:** Stomatology Hospital, School of Stomatology, Zhejiang University School of Medicine, Clinical Research Center for Oral Diseases of Zhejiang Province, Key Laboratory of Oral Biomedical Research of Zhejiang Province, Cancer Center of Zhejiang University, Hangzhou, China

**Keywords:** periodontitis, cardiovascular disease, bibliometric analysis, inflammation, periodontal pathogens

## Abstract

**Background:**

Periodontitis (PD) and cardiovascular diseases (CVD) rank among the most prevalent pathologies worldwide, and their correlation has been a subject of prolonged investigation. Numerous studies suggest shared etiological factors; however, a definitive causal connection remains unestablished. The objective of this study was to employ bibliometric and visual analyses in order to comprehensively examine the overarching characteristics, focal areas of research, and prospective trends pertaining to the PD-CVD relationship.

**Methods:**

We sourced articles, reviews, and online publications on PD- and CVD- research from the Web of Science Core Collection (WoSCC) spanning from January 1, 1993, to May 15, 2023. A triad of analytical tools (R-Bibliometrix, VOSviewer 1.6.19, and CiteSpace 6.2.R3) were utilized to facilitate collaboration network analysis, co-citation analysis, co-occurrence analysis, and citation burst detection.

**Results:**

Out of the 1,116 publications that fulfilled the eligibility criteria in the WoSCC database, the comprehensive characteristics analysis divulged a sustained growth trend in publication frequency. In the cluster analysis of reference co-citation and keyword co-occurrence, prominent themes such as “periodontitis”, “cardiovascular diseases”, “inflammation”, “*Porphyromonas gingivalis*”, and “atherosclerosis” consistently emerged. Contemporary topics such as “peri-implantitis,” “COVID-19”, “cardiovascular risk factors,” and “endocarditis” were pinpointed as burgeoning research hotspots.

**Conclusion:**

Based on this bibliometric study, in the field of association studies between PD and CVD, the etiologic mechanisms of both diseases have been intensively studied in the last three decades. Periodontal pathogens might serve as potential initiating factors linking PD and CVD. Inflammation may constitute a significant etiological factor shared by both diseases. Several emerging topics, such as COVID-19 and peri-implantitis, exhibit promising potential. This exhaustive overview casts light on pivotal research arenas, augmenting the field's understanding and stimulating further scholarly investigations.

## 1. Introduction

Cardiovascular disease (CVD) is a class of diseases involving the heart and blood vessels. It is also commonly known as heart and vascular disease (1). CVD encompasses a range of conditions that can affect the heart and blood vessels, including coronary artery disease, stroke, heart failure, arrhythmias, valvular heart disease, etc. However, periodontitis (PD), a severe form of gum disease, has been found to affect cardiovascular disease potentially. The link between periodontitis and cardiovascular disease has been explored through various studies and research efforts (2, 3). Both periodontitis and CVD share common risk factors such as smoking, diabetes, obesity and age. Moreover, periodontitis was found to be significantly correlated with heart failure, myocardial infarction, endocarditis, acute coronary syndrome, and atherosclerosis.

While more compelling and indicative evidence has come forth, emphasizing a potential causal association between these two pathologies, further research is imperative to definitively establish PD as an independent risk factor for CVD (4). Furthermore, an array of potential mechanisms have been explored in previous studies. Endothelial dysfunction, systemic inflammation and immune response, including C-reactive protein, interleukins, thrombotic markers and antibodies, have all been observedin patients with PD and CVD. Nonetheless, no study has definitively elucidated the precise mechanism that directly links PD to CVD. Periodontal pathogens (*Porphyromonas gingivalis Aggregatibacter actinomycetemcomitans, Tannerella forsythia, Fusobacterium nucleatum* and *Treponema denticola*) and their byproducts such as hemagglutinins, lipopolysaccharides (LPS), and gingipains were also found to contribute to the development of CVD (5). However, it is difficult to determine what kind of pathogens' byproducts play a vital role in the process. LPS, released by periodontal pathogens could activate TLR4/NF-*κ*B inflammatory pathway which is associated with CVD, could also derive from the large intestine (6). Based on the above issues, a better understanding of the PD and CVD relationship and a well-designed method are needed to clarify the complex network.

Bibliometrics, the quantitative study of scientific literature and research output, can play a valuable role to provide with visual representation of impact and influence of the current research. Compared with literature reviews and systematic reviews, bibliometrics offers objective data analysis with scalability and efficiency. It reduces the potential bias introduced by subjective decision-making. By analyzing publication trends over time, bibliometrics can identify emerging research areas and hot topics in specific fields. This can be particularly useful for researchers to stay updated with the latest developments and plan their strategies accordingly. As far as we know, there have been no Bibliometric analyses that can specifically on the associations between periodontal disease and various cardiovascular diseases. This bibliometric analysis is to analyze the overall features, research hotspots, and future research trends of the links between PD and CVD. Combining bibliometrics, experimental studies, clinical trials, and other research methods can contribute to a more comprehensive understanding of the relationship between periodontitis and cardiovascular disease.

## 2. Methods

### 2.1. Data source and search strategy

**Figure 1:**
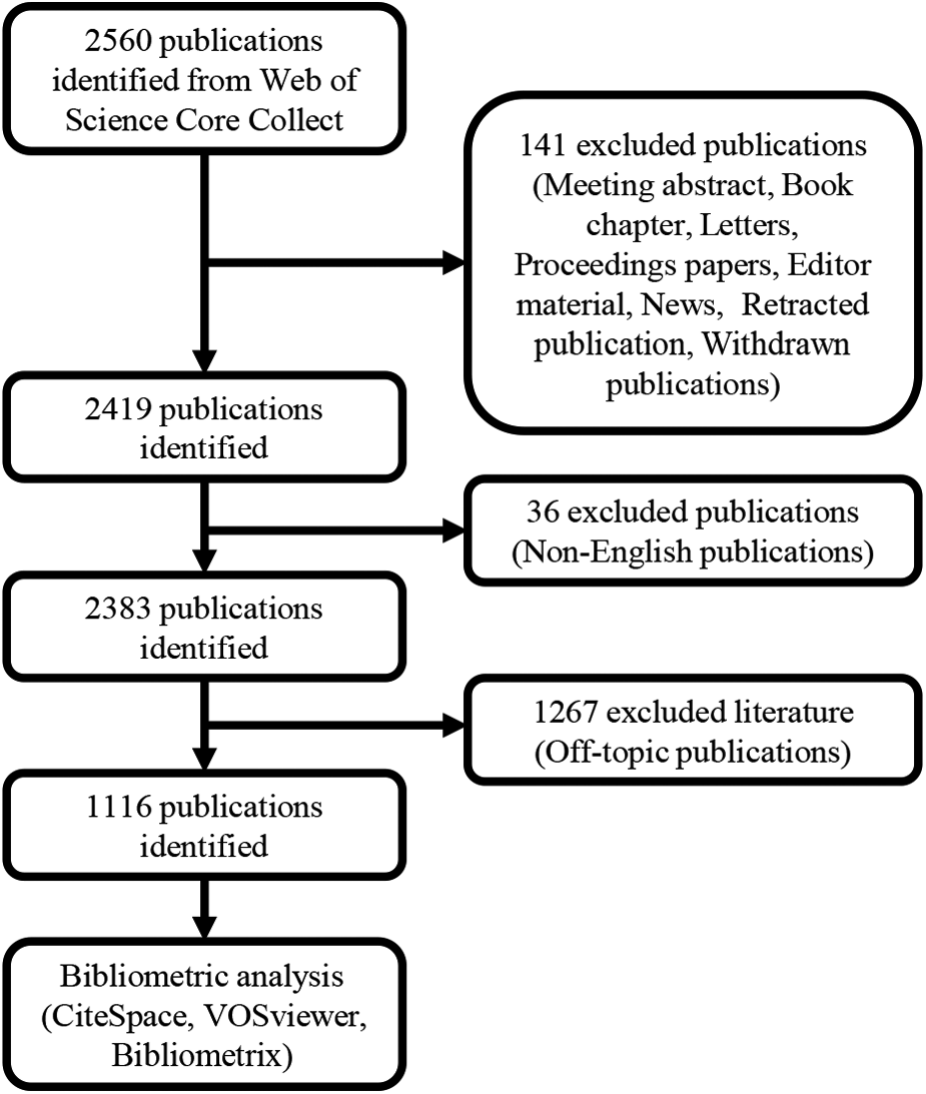
The flow chart of the bibliometrics study.

The subsequent search terminologies were employed to retrieve relevant literature from the Web of Science Core Collection (http://apps.webofknowledge.com/) on May 15, 2023: ([ALL = (heart)] OR ALL = (Cardiovascular)) AND ALL = (periodontitis). The search interval was fixed from 1993 to 2023 to ensure a comprehensive collection of pertinent literature. Data procurement was completed within a single day to circumvent potential variability due to daily database modifications. The selected publications were predominantly in English, with a focus on “articles”, “reviews”, and “online publications”. Prior to a meticulous assessment of the articles' titles and abstracts to ascertain if the literature conformed to the thematic focus, publications that did not adhere to the language and article type criteria were meticulously excluded. The search strategy yielded a total of 2,383 publications. Subsequently, the titles, abstracts, and keywords of the obtained papers were carefully reviewed by two independent reviewers (Kuangyun T. and Yongjia W.), to assess their relevance to the topic. Any disagreements between the two reviewers were resolved by discussing with another reviewer (Xuepeng C.) In this process, publications meeting the following criteria will be excluded:

Titles, abstracts, and keywords do not contain PD-related terms (Supplementary S3); titles, abstracts, and keywords do not contain CVD-related terms (Supplementary S3); and titles, abstracts, and keywords contain both PD-related and CVD-related terms, but the themes are unrelated to the topic “Links between PD and CVD” after reviewers' manual screening. Consequently, a corpus of 1,116 publications was compiled. These publications, consisting of full records with cited references, were exported as plain text files and archived in the format of download_.txt. A flowchart delineating the sequence of the bibliometric study is depicted in Figure 1.

### 2.2. Bibliometric analysis

Cite Space 6.2. R3 (Drexel University, USA) was deployed to conduct a comprehensive analysis of the incorporated papers, generating multiple visual outputs, such as dual-map overlay of journals, the network map of countries and institutions, co-cited references, reference co-citation clusters, and references with the strongest citation burst, and keywords with the strongest citation burst.

VOS viewer 1.6.19 (Leiden University, Holland) was instrumental in crafting the cooperation network of countries, institutions, authors, and co-cited authors and journals. This software enabled a cluster analysis and generated a network map along with a density map for high-frequency keywords. Within the visual map, varying nodes signified distinct entities such as countries, institutions, authors, and so forth. The size of a node was representative of the frequency or numbers associated with the entity, while the color of the node and the interconnecting line differentiated the clusters. The thickness of these lines signified the strength of the linkage between nodes.

R-Bibliometrix (University of Naples Federico, Italy) was employed to analyze the evolution trend of the topic over time according to the keywords, generate a network map depicting the global distribution of countries, and conduct a descriptive analysis of the publishing characteristics of journals. In tandem, the bibliometric online platform (https://bibliometric.com/) was also harnessed to depict the cooperation networks among countries.

## 3. Results

### 3.1. Publication outputs

**Figure 2:**
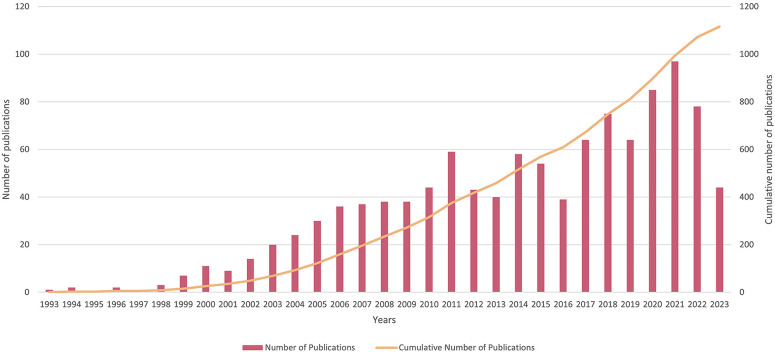
Publication years of the association between periodontitis and cardiovascular diseases.

A comprehensive review yielded a total of 1116 English-language articles. The seminal investigation into the correlation between periodontitis and heart diseases was first published by Destefano F in 1993 (7). Annual publication volumes remained below ten until the turn of the millennium (Figure 2). Thereafter, the rate of publication per annum began to accelerate notably. During the period spanning 2011–2017, the annual tally of publications experienced some volatility but consistently hovered within the bracket of 40 to 60 articles. Subsequent to 2017, the volume of published articles consistently breached the 60-article threshold, sustaining an average annual count of approximately 77 articles. This trend reached its apogee in 2021, with a remarkable annual yield of 97 articles.

### 3.2. Journals and co-cited journals

**Figure 3:**
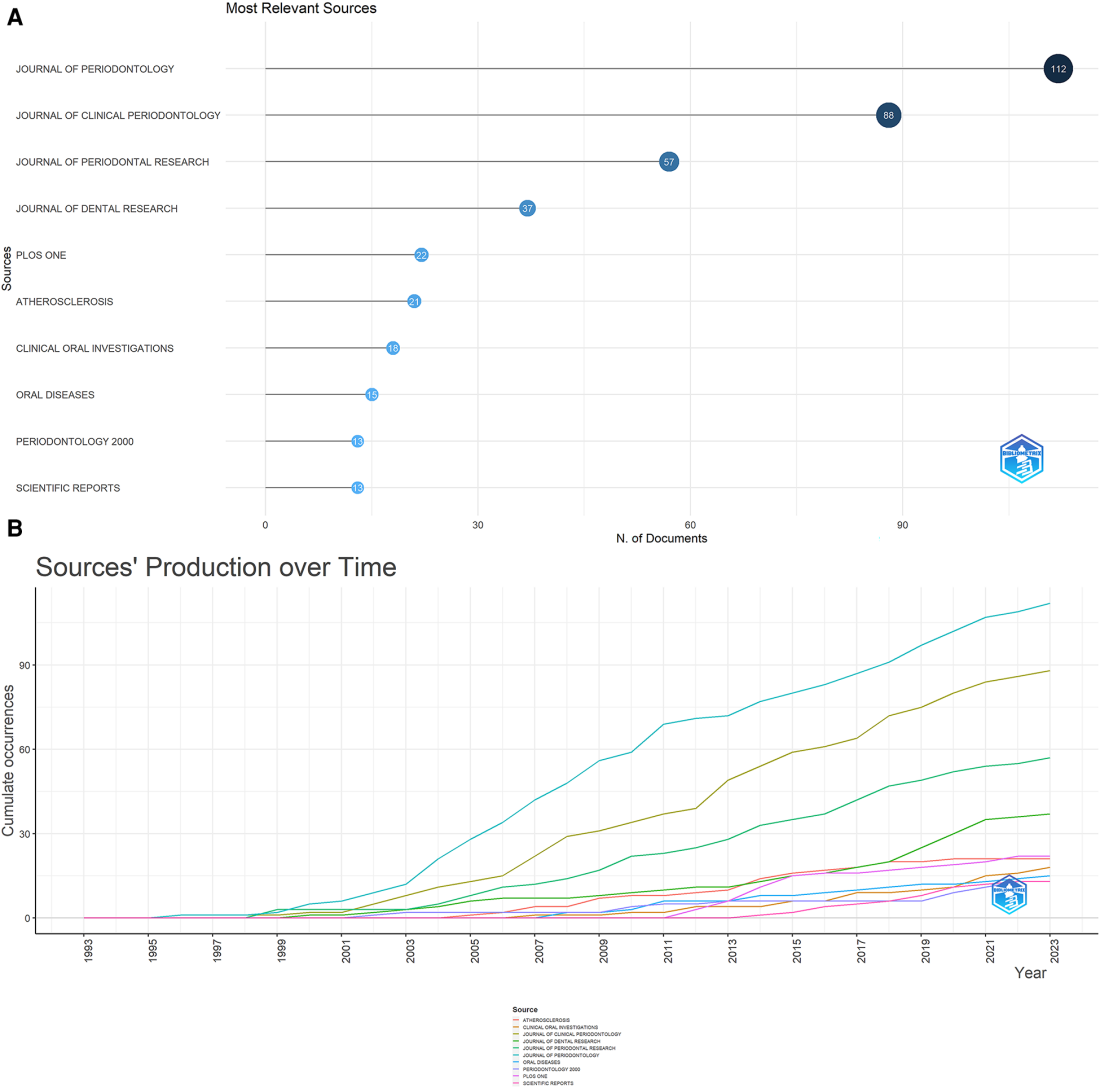
(**A**) The top 10 journals with the most publications generated by R-bibliometrix. (**B**) Annual occurrences of the top 10 journals with the most publications generated by R-Bibliometrix.

Journals often cited together by other scholars are called co-cited journals. Four hundred sixteen journals published 1,116 papers. Information on the top 10 and co-cited journals are presented in Table 1. As illustrated in Figure 3A, the top 10 journals accounted for 396 publications, equivalent to 35.48% of the total output. JOURNAL OF PERIODONTOLOGY led the field, having published 112 articles (10.04% of total), trailed by JOURNAL OF CLINICAL PERIODONTOLOGY (88, 7.88%), JOURNAL OF PERIODONTAL RESEARCH (57, 5.11%), JOURNAL OF DENTAL RESEARCH (37, 3.32%), and PLOS ONE (22, 1.97%). Among these ten journals, seven were based in the USA, two in Germany, and one in Ireland. Furthermore, four of these journals boast average impact factors exceeding 5.0.

**Table 1:** The top 10 journals and co-cited journals of the association between periodontitis and CVD.

Rank	Journal	*N* (%)	Country	IF (2022)	Co-Cited Journal	Co-Citation	Country	IF (2022)
1	J PERIODONTOL	10.04	USA	4.3	J PERIODONTOL	5,484	USA	4.3
2	J CLIN PERIODONTOL	7.88	USA	6.7	J CLIN PERIODONTOL	4,273	USA	6.7
3	J PERIODONTAL RES	5.11	USA	3.5	CIRCULATION	2051	USA	37.8
4	J DENT RES	3.32	USA	7.6	J DENT RES	1,910	USA	7.6
5	PLOS ONE	1.97	USA	3.7	J PERIODONTAL RES	1,452	USA	5.11
6	ATHEROSCLEROSIS	1.88	IRELAND	5.3	PERIODONTOL 2000	1,249	USA	18.6
7	CLIN ORAL INVEST	1.61	GERMANY	3.4	INFECT IMMUN	1,144	USA	3.1
8	ORAL DIS	1.34	USA	3.8	ARTERIOSCL THROM VAS	1,064	USA	8.7
9	PERIODONTOL 2000	1.16	USA	18.6	NEW ENGL J MED	886	USA	158.5
10	SCI REP-UK	1.16	GERMANY	4.6	ATHEROSCLEROSIS	884	IRELAND	5.3

Figure 3B traces the temporal evolution of annual publications from the ten most prolific journals. A striking surge in the publication volume from the JOURNAL OF PERIODONTOLOGY has been observable from 1998 onwards, firmly establishing its position at the vanguard of the field. Commencing in 2001, a steady increment in the publication counts of both the JOURNAL OF CLINICAL PERIODONTOLOGY and JOURNAL OF PERIODONTAL RESEARC has been seen, aligning them closely behind the Journal of Periodontology. And an ascending trend is also evident in the output of the remaining journals, most notably since 2011. As shown in Table 1, eight of the top co-cited journals amassed more than 1,000 co-citations. Nine of these ten journals originate from the USA, with half of them boasting impact factors exceeding 7.0. The upper echelon of co-cited journals includes J PERIODONTOL (5,484 co-citations), J CLIN PERIODONTOL (4,273 co-citations), CIRCULATION (2,051 co-citations), J DENT RES (1910 co-citations), and J PERIODONTAL RES (1,452 co-citations). Supplementary Figure S1 presents a network visualization diagram of the journal co-citation analysis, rendered using the VOS viewer. This map includes 77 journals, each of which has garnered over 100 citations.

**Figure 4:**
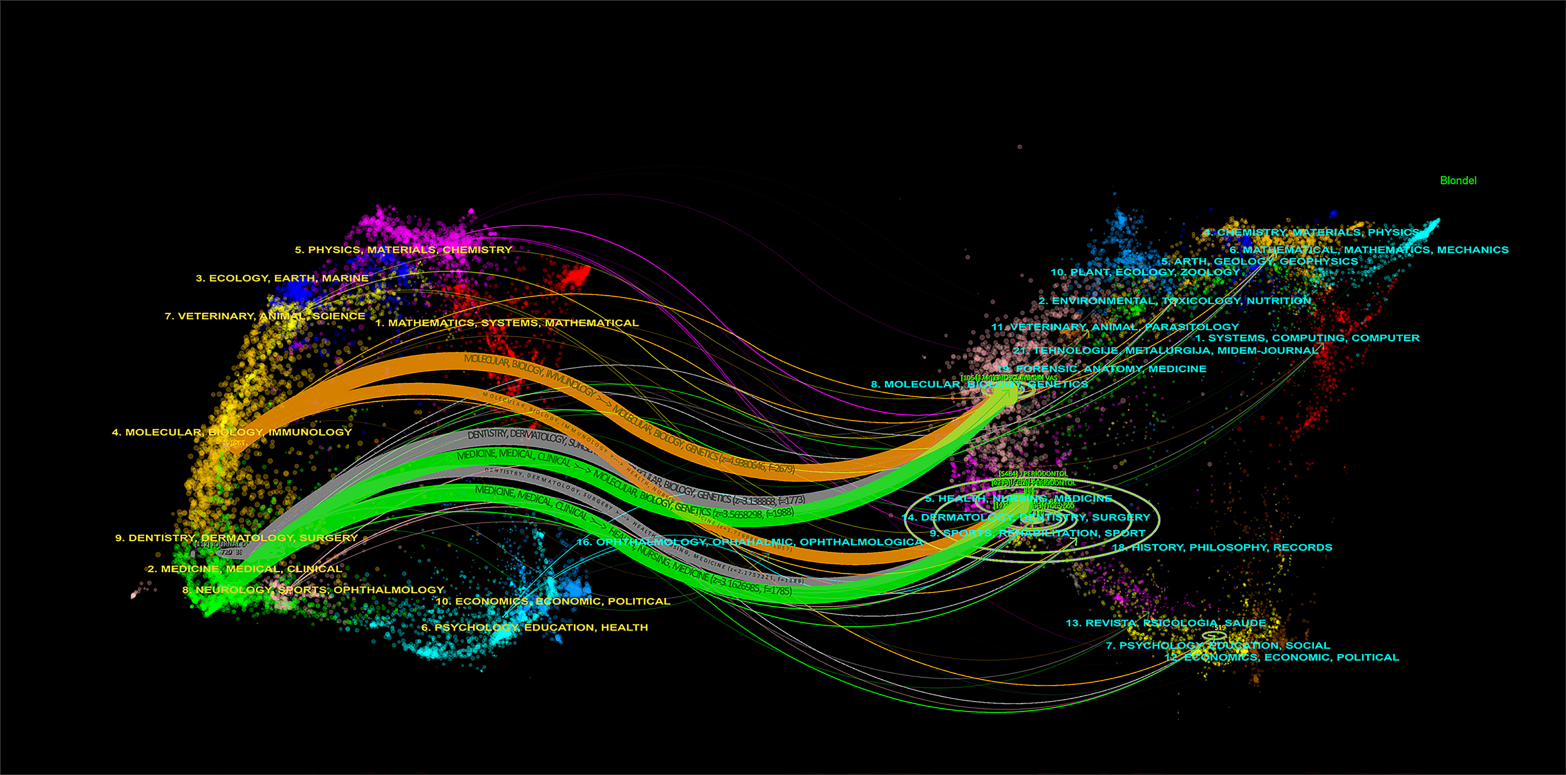
The dual-map overlay of journals related to the association between periodontitis and cardiovascular diseases.

The dual-map overlay of journals, as presented in Figure 4, elucidates the overarching scientific contribution in this field. The left side of the figure showcases a map of the citing journals, while the right side denotes a map of the cited journals. The label describes the subject covered by the journal. Colored line paths symbolize citation relationships, originating from the citing map and converging on the cited map to depict the trajectory of knowledge citations and the flow of information. For example, six main citation paths were shown in the current map, suggesting that the citing papers concerning Periodontitis and heart diseases primarily focused on journals in molecular, biology, immunology, medicine, medical, clinical, dentistry, dermatology, and surgery. At the same time, most of the cited articles were published in molecular, biology, genetics, health, nursing, and medicine.

### 3.3. Countries and institutions

**Figure 5:**
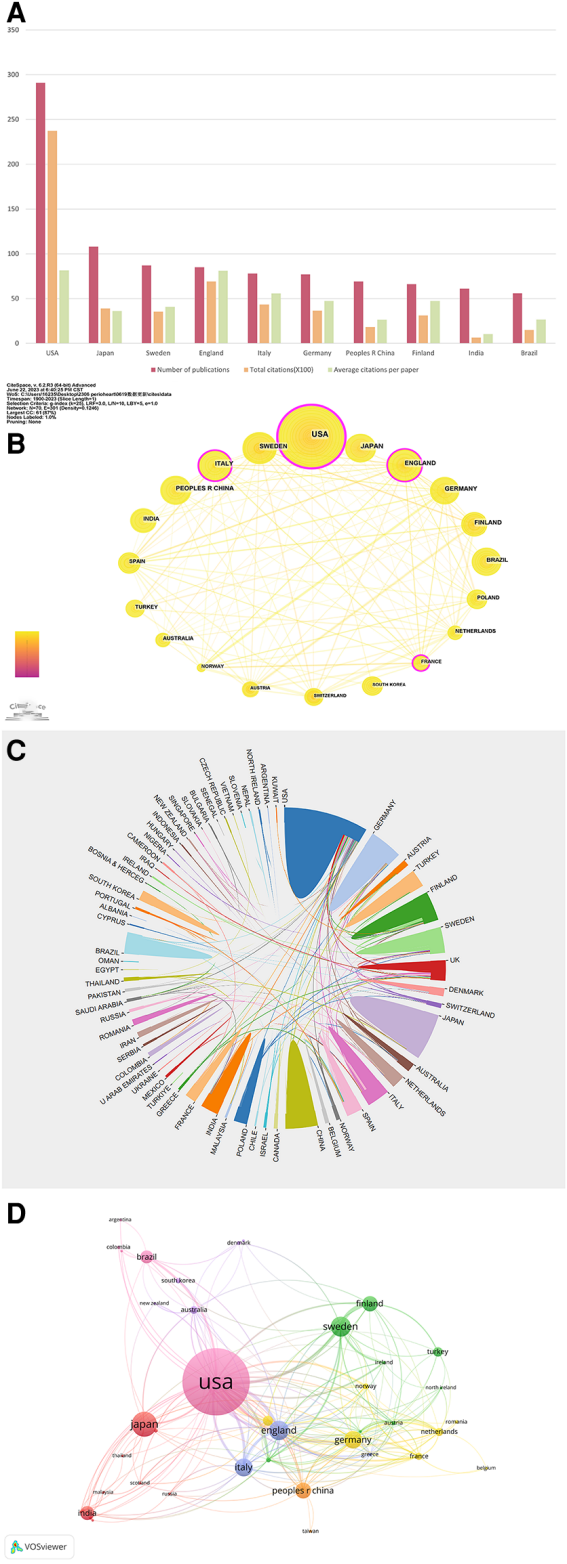
(**A**) The number of publications, total citations ( × 100), average citation per paper, and H-index of the 10 most productive countries/regions. (**B**) The country cooperation network of the top 20 productive countries generated by CiteSpace. (**C**) The international cooperation networks between countries. Line thickness between countries reflects the intensity of the closeness. (**D**) The network map for countries created by the VOS viewer.

A total of 70 countries have made contributions to the papers included. As delineated in Table 2 and Figure 5A, USA leads in terms of the number of publications (*n* = 291, accounting for 26.08%), followed by Japan (*n* = 108, accounting for 9.68%) and Sweden (*n* = 87, accounting for 7.80%). Regarding total citations, it can be seen that the USA had the most significant number of total citations (*n* = 23,736), followed by England (*n* = 6,900). When evaluating the average number of citations per paper among the top ten countries, Greece takes the lead with an average of 124.79 citations, with Australia and Jordan following closely, averaging 121.97 and 108.50 citations per paper respectively. Figures 5B,C depict the inter-country communication and collaboration. Figure 5B generated by CiteSpace, represents a network map of the top 20 most productive countries in this field of research: The node size is directly proportional to the number of publications, and the thickness of lines connecting the nodes represents the strength of the collaboration between the countries. We found that in the top 20 countries, the USA, England, and Germany were the core countries of the network map, indicating their extensive and robust collaborative efforts with other nations. The USA, in particular, has established productive collaborations spanning several continents. Some European countries (e.g., Finland, Sweden, and England) have established extensive and close cooperative relation. In contrast, intra-Asian communication appears relatively underdeveloped. Moreover, the betweenness centrality (BC) values reflect the significance of nodes within the collaborative network. Four nodes with BC values exceeding 0.1, namely the USA, England, Italy, and France, are highlighted with purple outer circles, signifying their pivotal roles in the network. Figure 5D is a network map generated by VOS viewer, featuring countries with more than five publications. This map consists of 42 nodes and 226 links, grouping into seven distinct clusters. Nodes within the same cluster indicate close cooperation. A global distribution map (Supplementary Figure S2) further illustrates international collaboration in Periodontitis and heart disease research. The link thickness between two countries represents the strength of their cooperation. The USA has the closest collaboration with England, Sweden, Italy, Brazil, and Switzerland, constituting the most significant transcontinental network. Notably, many European nations such as Finland, Sweden, Ireland, Germany, and the Netherlands also exhibit tight cooperation. Similarly, Asian countries like India, Saudi Arabia, and the United Arab Emirates demonstrate strong intra-regional collaboration. However, the level of collaboration between these Asian countries and European nations remains limited.

**Table 2:** The top 10 countries and institutions contributed to publications on the association between periodontitis and CVD [*n* (%)].

Rank	Country	*N* (%)	Institute	*N* (%)	Country
1	USA	291 (26.08%)	Univ Helsinki	64 (5.74%)	Finland
2	Japan	108 (9.68%)	Karolinska Inst	42 (3.76%)	Sweden
3	Sweden	87 (7.80%)	Univ N Carolina	39 (3.50%)	USA
4	England	85 (7.62%)	Tokyo Med & Dent Univ	31 (2.78%)	Japan
5	Italy	78 (6.99%)	UCL	25 (2.24%)	England
6	Germany	77 (6.90%)	Boston Univ	23 (2.06%)	USA
7	Peoples R China	69 (6.18%)	Univ Amsterdam	23 (2.06%)	Netherlands
8	Finland	66 (5.91%)	Vrije Univ Amsterdam	23 (2.06%)	Netherlands
9	India	61 (5.46%)	Univ Oslo	22 (1.97%)	Norway
10	Brazil	56 (5.02%)	Univ Tokyo	22 (1.97%)	Japan

**Figure 6:**
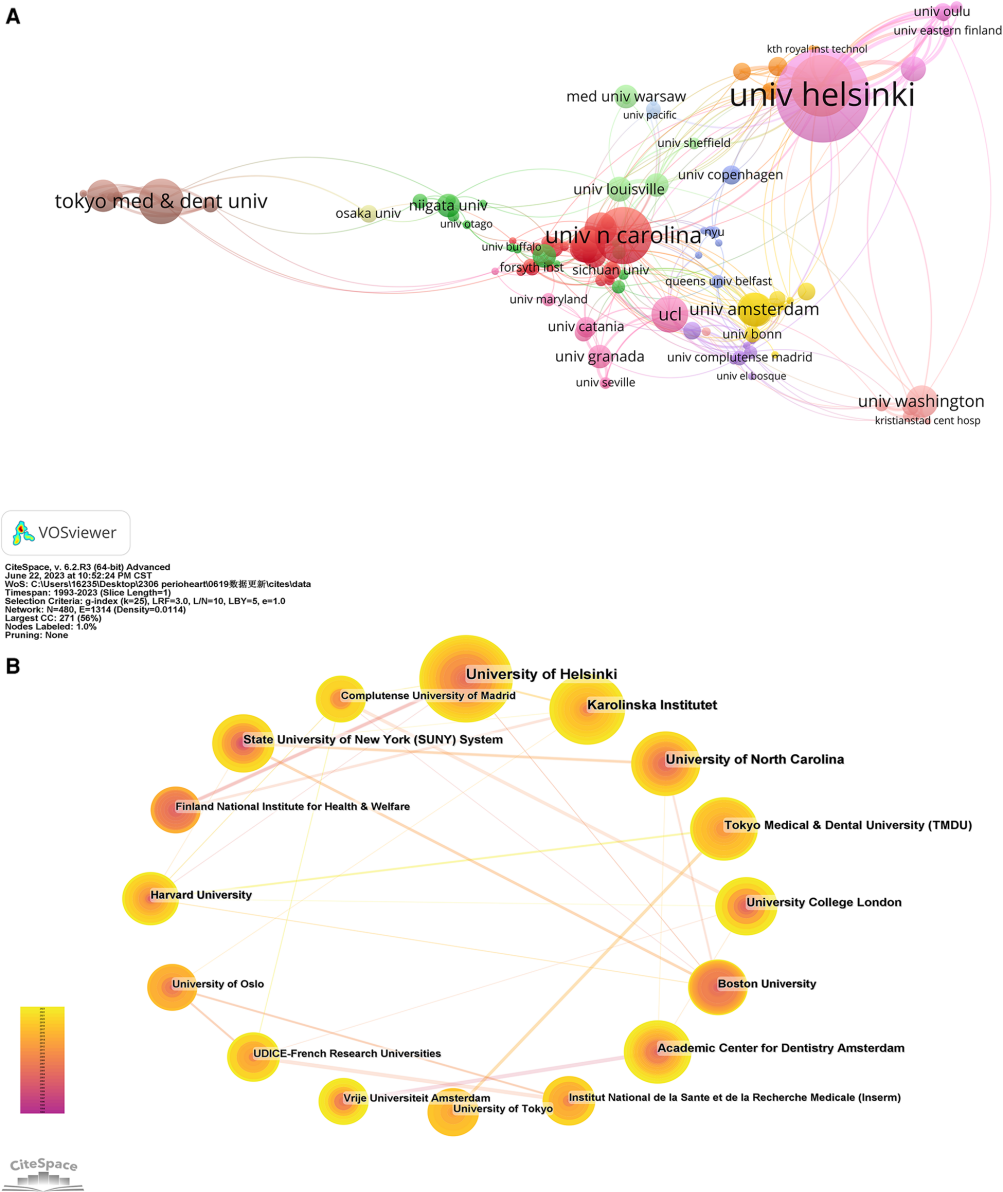
(**A**) The network map of institutions for the association between periodontitis and cardiovascular diseases was created by VOSviewer. (**B**) The cooperation network of the top 20 institutions generated by CiteSpace.

A total of 1,407 institutions have contributed to the body of 1,116 papers. Table 2 lists the top ten institutions, which collectively account for 314 articles, equivalent to 28.14% of the total output. Univ Helsinki tops this list with 64 articles (5.74%), followed by Karolinska Inst with 53 (3.76%), Univ N Carolina with 41 (3.50%), Tokyo Med & Dent Univ with 37 (2.78%) and Ucl with 38 (2.24%). VOS viewer generated a cooperation network of institutions, as depicted in Figure 6A. This network encompasses 112 nodes and 406 links, representing institutions with a collaborative frequency exceeding five. These 112 institutions aggregate into 13 distinct clusters, indicating particularly active collaboration within each cluster. Moreover, Cite Space was employed to render a cooperation network for the top 16 institutions. As indicated in Figure 6B, several collaborations exist among these leading institutions.

### 3.4. Authors and co-cited authors

**Figure 7:**
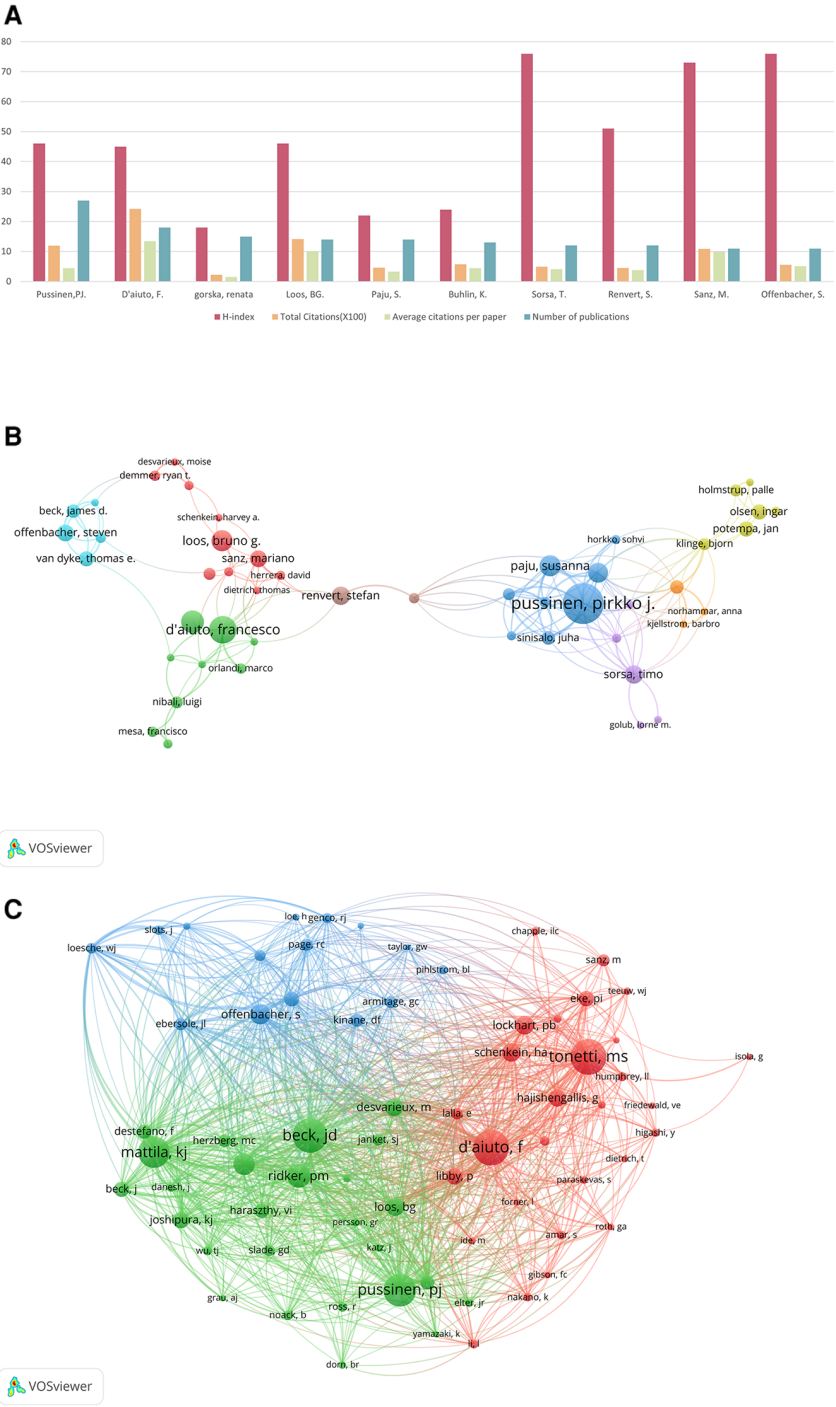
(**A**) The total citations ( × 100), average citation per paper ( × 10), and H-index of the top 10 most prolific authors. (**B**) The network map of authors for the association between periodontitis and cardiovascular diseases. (**C**) Author co-citation analysis by VOSviewer.

Network maps of authors and co-cited authors were generated using VOSviewer to illuminate influential research groups and authors. From the identified 1,116 articles, a total of 5,346 authors were cataloged. The top 10 authors and co-cited authors are detailed in Table 3. Figure 7A presents a tripartite analysis of the top ten most productive authors, considering their total citations ( × 100), average citations per paper ( × 10), and H-index. This index measures both the productivity and citation impact of an author's publications. These ten most productive authors published 147 (13.17%) papers. Among them, Pussinen, Pirkko J. (27, 2.42%) had the most publications, closely followed by D'aiuto, Francesco (18, 1.61%), and Gorska, Renata (15, 1.34%). The citation analysis, illustrated in Figure 7A, shows that D'aiuto, Francesco's work has been cited the most frequently (2,419 citations), with an impressive average of 134.39 citations per paper and an H-index of 46. Offenbacher, Steven, while ranking sixth in total citations (558) and fourth in average citations per paper (50.73), boasts an exceptional H-index of 76. Sorsa, Timo, with 491 total citations and an average of 40.92 citations per paper, also achieves a high H-index of 76. Nine of these authors boasted an H-index exceeding 20. Figure 7B showcases a network map of authors who contributed more than five papers, revealing a lack of close collaboration among these authors. Further, Figure 7C features a co-citation network map of authors, identifying 69 authors with over 80 citations each as influential researchers. Node size corresponds to the citation frequency while connecting lines signify cooperation between authors; the line thickness corresponds to the strength of these connections. The total link strength (TLS), which reflects the influence of an author's published articles on other researchers within this field, reveals that D'aiuto, Francesco holds the highest TLS at 8,072, followed by Beck, James D. (7,279) and Pussinen, Pirkko J (6,412).

**Table 3:** The top 10 authors and co-cited authors of the association between periodontitis and CVD.

Rank	Author	*N* (%)	Citations	H-Index	Co-Cited Author	Citations	Total Link Strength
1	Pussinen, PJ	27 (2.42%)	1,193	46	Tonetti, MS	446	5,812
2	D'aiuto, F	18 (1.61%)	2,419	45	D'aiuto, F	442	8,072
3	Gorska, R	15 (1.34%)	225	18	Beck, JD	431	7,279
4	Loos, BG	14 (1.25%)	1,413	46	Pussinen, PJ	404	6,412
5	Paju, S	14 (1.25%)	458	22	Mattila, KJ	392	6,386
6	Buhlin, K	13 (1.16%)	574	24	Ridker, PM	291	4,996
7	Sorsa, To	12 (1.08%)	491	76	Hujoel, PP	268	4,516
8	Renvert, S	12 (1.08%)	454	51	Offenbacher, S	252	4,378
9	Sanz, M	11 (9.86%)	1,085	73	Lockhart, PB	231	2,799
10	Offenbacher, S	11 (9.86%)	558	76	Schenkein, HA	228	3,314

### 3.5. Co-cited references and the citation bursts

**Figure 8:**
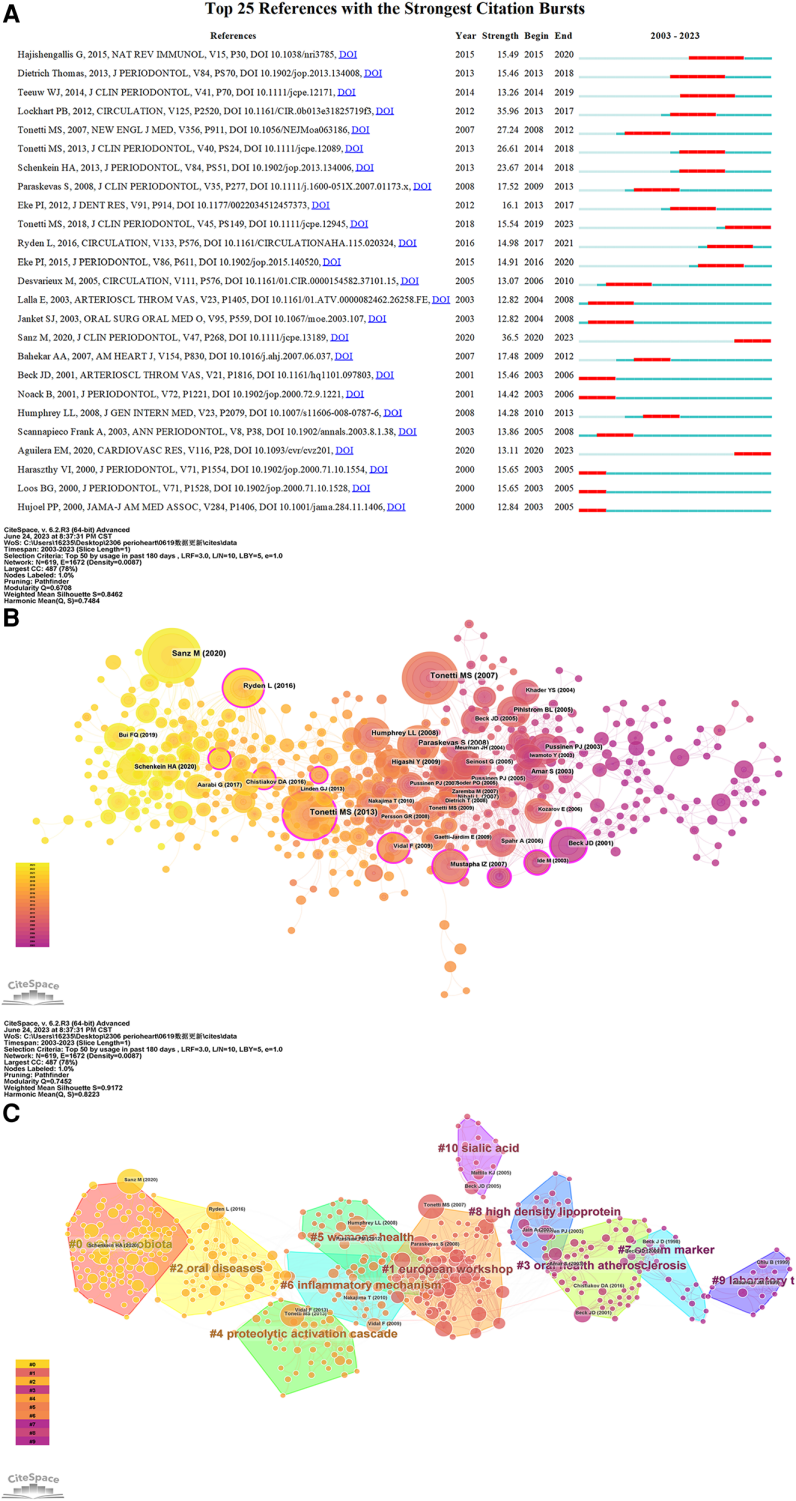
(**A**) The top 25 references with the strongest citation bursts in the co-citation network. (**B**) The network map of reference co-citation for the association between periodontitis and cardiovascular diseases. (**C**) The network map of reference co-ciation clusters for the association between periodontitis and cardiovascular diseases.

References cited by a series of articles are determined as co-cited references. Table 4 presents the top ten co-citations relating to periodontitis and heart diseases, each garnering no fewer than 50 citations. The most frequently cited reference accrued 187 co-citations. References with citation bursts are those papers that enjoy a surge of citations over a certain period. In CiteSpace, the k value for the g-index was configured at 5, and the minimum duration of the burst was defined as two years Consequently, we identified twenty-five references with significant citation bursts over the past two decades (Figure 8A). In Figure 8A, a citation burst period is demarcated by a red line, which signifies the start and end years of the burst duration. Citation bursts were first observed in 2000, with the strongest burst associated with two papers published in 2013 and 2015. Approximately 52% of the references experienced citation bursts between 2013 and 2023, with the most recent burst occurring in 2020. We also used CiteSpace to create a network map of co-cited references. Figures 8B,C depict nodes representing the references, which are grouped into 11 specific clusters such as “#0 dental health”, “#1 ankle-brachial index”, “#2 rheumatoid arthritis”, and “#3 stroke”.

**Table 4:** Top 10 co-cited references related to the association between periodontitis and CVD.

Rank	Co-Cited Reference	Co-Citation
1	Beck J, 1996, J PERIODONTOL, v67, p1123, DOI 10.1902/jop.1996.67.10s.1123	187
2	Tonetti MS, 2007, NEW ENGL J MED, v356, p911, DOI 10.1056/nejmoa063186	186
3	Destefano F, 1993, BRIT MED J, v306, p688, DOI 10.1136/bmj.306.6879.688	171
4	Haraszthy VI, 2000, J PERIODONTOL, v71, p1554, DOI 10.1902/jop.2000.71.10.1554	169
5	Lockhart PB, 2012, CIRCULATION, v125, p2520, DOI 10.1161/cir.0b013e31825719f3	161
6	Mattila LJ, 1989, BRIT MED J, v298, p779, DOI 10.1136/bmj.298.6676.779	151
7	Loos BG, 2000, J PERIODONTOL, v71, p1528, DOI 10.1902/jop.2000.71.10.1528	131
8	Bahekar AA, 2007, AM HEART J, v154, p830, DOI 10.1016/j.ahj.2007.06.037	118
9	Humphrey LL, 2008, J GEN INTERN MED, v23, p2079, DOI 10.1007/s11606-008-0787-6	115
10	Beck JD, 2001, ARTERIOSCL THROM VAS, v21, p1816, DOI 10.1161/hq1101.097803	107

### 3.6. Keywords

**Figure 9:**
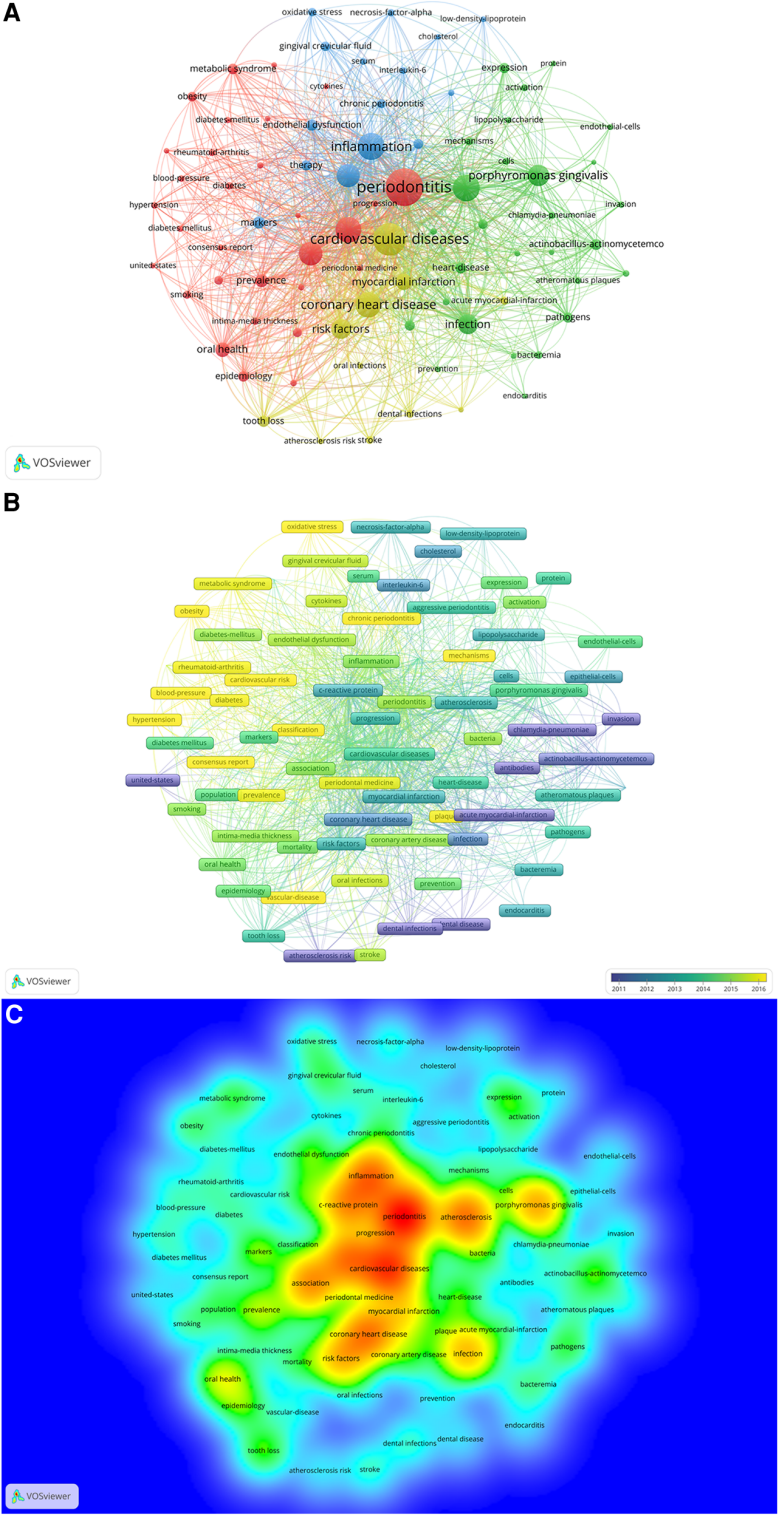
(**A**) The network map of keywords for the association between periodontitis and cardiovascular diseases generated by VOSviewer. (**B**) Overlay visualization of keywords generated by VOSviewer. (**C**) The density map of keywords for the association between periodontitis and cardiovascular diseases by VOSviewer.

Figure 9A presents a keyword network map created by VOSviewer, featuring keywords that occurred more than 20 times. From the 1,116 articles, we extracted 3,515 keywords and applied a frequency threshold of at least 20. Keywords with similar meanings were consolidated, yielding 86 keywords for analysis. The overlay visualization map of these 86 keywords is depicted in Figure 9B. The blue nodes represent early-stage keywords, while the yellow nodes denote the most recently emerged ones. Initially, keywords such as “acute myocardial-infarction”, “atherosclerosis risk”, “*chlamydia-pneumoniae*”, and “dental infections” dominated. Recently, however, “chronic periodontitis”, “mechanisms”, “cardiovascular risk”, “periodontal medicine”, and “oxidative stress” have emerged as the primary topics. As revealed in Figure 9C and Table 5, “periodontitis” was the most significant keyword, with 567 co-occurrences, followed by cardiovascular diseases (443), inflammation (323), atherosclerosis (318), periodontal diseases (294), coronary heart disease (291), and association (255). The top 20 keywords encompassed terms related to periodontal disease (e.g., “periodontitis”, “tooth loss”), cardiovascular diseases (e.g., “heart diseases”, “atherosclerosis”, “coronary heart disease”, “myocardial infarction”), and disease mechanisms (e.g., “inflammation”, “infection”, “endothelial dysfunction”). Keywords, such as “*Porphyromonas gingivalis*” and “*actinobacillus-actinomycetemcomitans*”, denoted periodontal pathogens that may impact cardiovascular health.

**Table 5:** The top 20 keywords in terms of frequency for the field of the association between periodontitis and CVD.

Rank	Keyword	*N* (%)	Rank	Keyword	*N* (%)
1	Periodontitis	567 (50.80%)	11	Risk factors	191 (17.11%)
2	Cardiovascular diseases	443 (39.70%)	12	Myocardial infarction	135 (12.10%)
3	Inflammation	323 (28.94%)	13	Oral health	110 (9.86%)
4	Atherosclerosis	318 (28.49%)	14	Prevalence	93 (8.33%)
5	Periodontal diseases	294 (26.34%)	15	Markers	80 (7.17%)
6	Coronary heart disease	291 (26.08%)	16	Tooth loss	74 (6.63%)
7	Association	255 (22.85%)	17	Endothelial dysfunction	71 (6.36%)
8	C-reactive protein	248 (22.22%)	18	Actinobacillus-actinomycetemcomitans	70 (6.27%)
9	Porphyromonas gingivalis	219 (19.62%)	19	Heart-disease	67 (6.00%)
10	Infection	193 (17.29%)	20	Epidemiology	66 (5.91%)

Cluster 1, the largest, contained 30 keywords including “periodontitis”, “metabolic syndrome”, “obesity”, among others. Cluster 2 encompassed 28 keywords predominantly related to “atherosclerosis”, “*Porphyromonas gingivalis*”, “infection”, and more. The 15 keywords in Cluster 3 included “inflammation”, “c-reactive protein”, “endothelial dysfunction”, etc., while Cluster 4 comprised 11 keywords, such as “cardiovascular diseases”, “coronary heart disease”, “myocardial infarction”, and others.

**Figure 10:**
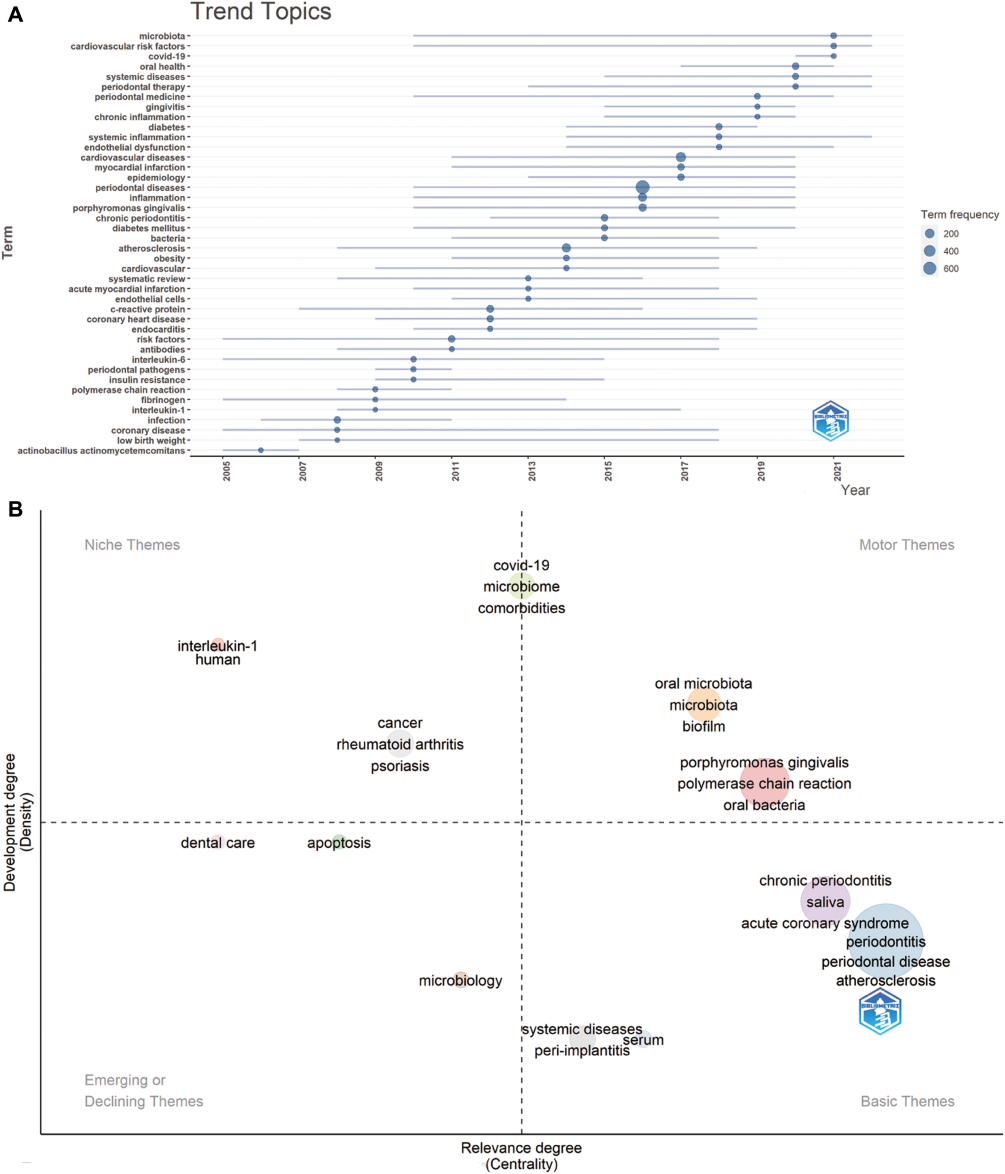
(**A**) Trend topics. The X-axis represents the year, while the Y-axis represents the cumulative keyword occurrences. (**B**) The keywords thematic map generated by R-Bibliometrix. The X-axis represents the centrality indicating the importance of a theme; The Y-axis symbolizes the density indicating the development of a theme.

Figure 10A illustrates a trend map, generated based on the occurrence frequency of author keywords, with a minimum word frequency set at 5 and three words displayed per year. The keywords, including “risk factors” and “coronary disease”, had the longest duration (13 years), followed by “microbiota” (12 years) and “cardiovascular risk factors” (12 years). “Covid-19” emerged in the field of periodontitis and CVD in 2020. In 2021, “microbiota”, “cardiovascular risk factors”, and “covid-19” displayed the highest frequency, while “oral health”, “systemic diseases”, and “periodontal therapy” dominated in 2020.

The thematic keyword map in Figure 10B, generated by R-Bibliometrix, analyzes 250 keywords with a minimum cluster frequency of 5 and three labels per cluster. Two high-density and centrality clusters in the upper right quadrant (motor theme) serve as central, well-developed themes for structuring periodontitis and heart diseases research, featuring keywords such as “oral microbiota” and “*Porphyromonas gingivalis*”. The upper-left quadrant (niche theme) presents two clusters: one with “interleukin-1” and “humans”, and another featuring “cancer”, “rheumatoid arthritis”, and “psoriasis”. Another cluster, exhibiting high density and moderate centrality, is positioned between the upper right and upper left quadrants, including “covid-19”, “microbiome”, and “comorbidities”. The third quadrant (emerging or declining theme) encompasses “dental care”, “apoptosis”, and “microbiology”. Lastly, the fourth quadrant (basic themes) hosts four clusters, including terms such as “systemic diseases”, “peri-implantitis”, “chronic periodontitis”, “saliva”, “acute coronary syndrome”, “periodontitis”, “periodontal disease”, “atherosclerosis”, and “serum”.

## 4. Discussion

This study presents a visualized analysis of the association between periodontitis and CVD, identifying several characteristic qualities of this research field. The examination is based on articles and reviews published within this field up to May 15, 2023. Data were sourced from the Web of Science Core Collection (WOSCC) and analyzed using CiteSpace, VOSviewer, and Bibliometrix to evaluate the current research landscape and emerging trends. The surge in published content indicates an escalating interest in the interplay between these two diseases, with dentistry emerging as the primary research domain. While periodontal disease-related journals represent the main platforms for publication, several highly-cited articles have also found their place in general scientific research journals. Analysis of countries, institutions, and authors suggests limited global collaboration, with the United States outpacing other nations in terms of research output.

In a paper, the judicious use of keywords enables accurate and rapid identification of a study's themes and topics. This principle is equally applicable to bibliometrics, where keyword analysis provides insights into research trends in a specific field. Table 5 exhibits the top 20 most frequent keywords, predominantly focusing on PD, cardiovascular diseases, disease mechanisms, periodontal pathogens, among others. Prominent keywords such as “inflammation”, “C-reactive protein”, “*Porphyromonas gingivalis*”, and “infection”, suggest that inflammation and oral infection are critical links between periodontitis and CVD, defining prevalent research directions. Our study incorporates a co-occurrence analysis of 86 keywords that appeared more than 20 times. As illustrated in Figure 9A, these keywords, represented as nodes, form a complex network where the size of a node corresponds to the keyword's frequency and indicates the primary discussion topics linking periodontitis and CVD. Furthermore, the emergence of four distinct clusters from these keywords reaffirms the aforementioned inference that both inflammation and oral infection are significant factors in the interplay between these two diseases.

Figure 8A indicates that the latest burst references are Sanz M (8) and Aguilera EM (9), with Hajishengallis G (10) yielding the highest burst citation strength of 15.49. The latter review discusses two potential mechanisms connecting periodontitis to cardiovascular disease. Initially, gingival ulceration within the periodontal pocket allows bacteria to enter systemic circulation, leading to bacteremia, a condition frequently observed in periodontitis patients that may provide an atherogenic stimulus. Alternatively, a mice study suggested that the bacteria *Porphyromonas gingivalis* could alter gut microbiota, indirectly inducing systemic inflammation (11). Sanz M's paper, the most recent reference to exhibit a strong citation burst in our study, is a consensus report on the PD-CVD connection. The paper concludes that the robust epidemiological evidence linking periodontitis to an increased risk of future atherosclerotic cardiovascular disease, affirms the biological plausibility of periodontitis's impact on CVD.

Regarding the keyword thematic map produced by R-Bibliometrix (Figure 10B), two clusters are prominent in the upper right quadrant, including “oral microbiota”, “*Porphyromonas gingivalis”*, and “biofilm”. These terms, distinguished by their high density and centrality, signal well-established and core research themes within the periodontitis and CVD interplay. Meanwhile, keywords like “saliva” and “peri-implantitis” situated in the third quadrant represent burgeoning topics that are yet to be fully explored, thus likely to be focal points in future research. Notably, a cluster straddling the right and left upper quadrants includes “COVID-19”, “microbiome”, and “comorbidities”, terms that have garnered increasing attention in recent years.

The top 20 most frequent keywords included“*Porphyromonas gingivalis*” and “infection”, while the top 50 keywords included “*Actinobacillus actinomycetemcomitans*”, “pathogens”, “bacteria”, “bacteremia”, “dental infections”, and “plaque”. As shown in the keyword thematic map, “oral microbiota”, “*Porphyromonas gingivalis*”, and “biofilm” were well-established and core research themes. Considering the above results, periodontal pathogens, especially *Porphyromonas gingivalis*, are likely potential initial factors for the connection to cardiovascular disease. The *Porphyromonas gingivalis*, a significant pathogen of periodontal diseases, has been implicated in the development and progression of several cardiovascular diseases. Here are some cardiovascular conditions that may be affected by *Porphyromonas gingivalis*, such as atherosclerosis, myocardial infarction, stroke and endocarditis. *Porphyromonas gingivalis*, can potentially affect cardiovascular diseases through various mechanisms (12, 13). First of all, *Porphyromonas gingivalis* can induce a localized inflammatory response in the gum tissues affected by periodontal diseases. The release of bacterial toxins and pro-inflammatory molecules by *Porphyromonas gingivalis* can lead to the production of inflammatory mediators such as cytokines and acute-phase proteins, including Tumor Necrosis Factor-alpha (TNF-alpha), Interleukin-1 beta (IL-1β), Interleukin-6 (IL-6), Interleukin-8 (IL-8) and Interleukin-17 (IL-17) (5, 14). These inflammatory mediators can enter the bloodstream and promote systemic inflammation throughout the body, including the blood vessels and arterial walls. Systemic inflammation can contribute to the development of atherosclerosis, plaque destabilization, and increased risk of cardiovascular events. Widen et al. found out that serum levels of Interleukin-8 had increased significantly in acute coronary syndrome (15). Furthermore, *Porphyromonas gingivalis* induced inflammation can negatively impact the endothelial cells that line the blood vessels, leading to endothelial dysfunction (16). Endothelial dysfunction is a crucial early step in the development of atherosclerosis, acute coronary syndrome and myocardial infarction. The bacterial toxins released by *Porphyromonas gingivalis* can impair the normal functioning of endothelial cells, affecting their ability to regulate vascular tone, promote vasodilation, and maintain proper blood flow (17). Belanger's study (18) showed that *Porphyromonas gingivalis* expressing hemagglutinin A, had an increased capability to adhere to and invade human coronary artery endothelial cells. Lastly, the immune response triggered by *Porphyromonas gingivalis* and its byproducts can contribute to the formation of atherosclerotic plaques (19). The bacterium can activate immune cells leading to their recruitment to the arterial walls affected by atherosclerosis. Peptidoglycan, one kind of byproduct of *P. gingivalis*, could elevate intercellular adhesion molecule-1 production to promote monocyte migration to the cardiac endothelium (20). These immune cells, along with *Porphyromonas gingivalis*-induced inflammation, promote the uptake of modified low-density lipoprotein (LDL) by macrophages, resulting in the formation of foam cells (21, 22). *P. gingivalis* promotes lipid accumulation in macrophages by upregulating CD36 and inhibiting cholesterol efflux from macrophages by promoting lysosomal integral membrane protein 2 (23, 24). By interfering with lipid metabolism, *P. gingivalis* promotes macrophages to covert to foam cells. Foam cells accumulate in the arterial walls and are a hallmark of early atherosclerotic lesions (25).

Furthermore, some interventional studies have investigated the impact of controlling periodontal pathogens on cardiovascular outcomes by periodontal treatment. While the results are mixed, some studies have shown that periodontal treatment, such as scaling and root planning, can lead to improvements in cardiovascular measures, including endothelial function and arterial stiffness. Caúla's research (26) showed that periodontal treatment was effective in reducing inflammatory markers related to the risk for cardiovascular disease in 6 months, such as C-reactive protein, cholesterol, and triglycerides. D'Aiuto et al. had similar conclusions in their studies (27). Periodontal treatment reduced inflammatory plasma levels, including C-reactive protein, interleukin-6 and tumor necrosis factor-alpha. Interestingly, Graziani et al. had found that full-mouth scaling and root planning were more effective in systemic inflammation reduction than multiple quadrant scaling and root plannings (28). Based on one of the most cited articles in our study, the American Heart Association supported an independent association between periodontitis and atherosclerotic cardiovascular disease (29). Therefore, substantial evidence has supported that periodontal bacteria could contribute to cardiovascular disease as an important potential independent risk factor.

The relationship between periodontal pathogens and cardiovascular disease is a complex and evolving area of research. While the exact mechanisms are not fully understood, several hypotheses have been proposed to explain how periodontal pathogens may influence the development and progression of cardiovascular disease, such as inflammation, bacteremia, endothelial dysfunction, immune response and autoimmunity, platelet activation, and clot formation, among others (4, 30). According to our study, the most frequent keywords related to the mechanisms were “inflammation” and “C-reactive protein,” whose appearance ratios in our 1,116 articles as keywords were 28.94% and 22.22%, respectively. These ratios were significantly higher than those of other keywords, such as “endothelial dysfunction” (6.36%), “oxidative stress” (3.76%), “lipopolysaccharide” (2.42%), and “antibodies” (2.15%). In general, the appearance ratios of keywords related to inflammation, including various inflammation mediators, were remarkably higher than those of other mechanism-related keywords. A growing body of literature suggests that inflammation might be the more likely way for periodontal pathogens to affect cardiovascular disease.

The role of inflammation is crucial in understanding the associations between periodontitis and cardiovascular disease. When periodontal tissues become inflamed, pro-inflammatory cytokines and other inflammatory mediators are released into the bloodstream (31). These inflammatory markers can directly affect the vascular endothelium. The systemic mediators of inflammation released from inflammatory periodontal tissues included C-reactive protein, interleukins and matrix metalloproteinases (32, 33). Besides inflammatory cytokines, antibodies could also mediate system inflammation. Antibodies can stimulate the immune response and trigger an inflammatory cascade (34). Hajishengallis' article summarized that, when antibodies recognize and bind to periodontal antigen, they can activate immune cells such as macrophages, neutrophils, and T cells to release pro-inflammatory molecules into blood vessels, promoting the development of atherosclerosis and contribute to cardiovascular risk (10). Pussinen, who had the most publications in our study, found that serum antibodies to *Actinobacillus actinomycetemcomitans* and *Porphyromonas gingivalis* were associated with coronary heart disease in a study containing 1,000 samples (35). Some studies suggested that antibodies produced against periodontal pathogens can cross-react with proteins present in cardiovascular tissues (36, 37). IgG anti–beta-1-adrenorecepters extracted from periodontal patients could work against rat cardiac membranes *in vitro*. Those patients with periodontitis who had IgG anti–beta-1-adrenorecepters had showed lower heart rate variability, which means impaired contractility of the heart (38, 39). When antibodies mistakenly recognize and target cardiovascular proteins due to their similarity to periodontal antigens, they can trigger an autoimmune-like response. This response can lead to chronic inflammation and contribute to the development of CVD. To sum up, studying how periodontal pathogens mediate the impact on cardiovascular disease through inflammation and inflammatory mediators is currently a research hotspot, and it holds great potential as a future research direction.

Moreover, certain genetic background can predispose patients to suffer from periodontitis and cardiovascular disease more easily than others. Teeuw WJ et al. (40) demonstrated a significant association between ANRIL and periodontitis, as well as an association between ANRIL variations and C reactive protein levels in periodontitis patients, further proving that genes are risk factors in both periodontitis and systemic inflammation.

Regarding the various cardiovascular diseases related to PD, the top 5 keywords included “atherosclerosis,” “coronary heart disease,” “myocardial infarction,” “coronary artery disease,” and “acute myocardial infarction” in our study. Atherosclerosis is the most common type of cardiovascular disease and a major cause of coronary heart disease, myocardial infarction, and other related complications. Epidemiologic studies have strongly associated periodontitis with atherosclerotic disease (41, 42). At least, 11 studies have reported positive associations between periodontitis and atherosclerotic cardiovascular disease (41, 42). Hayashida et al. reported a dose-dependent association between periodontal pocket depth and markers of atherosclerosis in 2013 (43). In Beukers' study, which included 60,174 cases, periodontitis was linked with acute cardiovascular disease with an odds ratio of 2.52. In another study, periodontitis was found significantly associated with the incident of acute myocardial infarction, with an odds ratio of 2.4 (2). Moreover, Górski et al. found that the extent of periodontitis was more strongly associated with acute myocardial infarction than the severity (44).

In addition to the previously mentioned epidemiological investigations, a multitude of biological plausibilities have been put forth to elucidate the connection between periodontal diseases and atherosclerosis (30). It is hypothesized that these bacteria may contribute to the formation and progression of atherosclerotic lesions. Available evidence has been presented and evaluated. The incidence rate of bacteremia can reach 49.4% after periodontal treatments (45). At the same time, the bacteria associated with periodontal diseases, such as *Porphyromonas gingivalis*, have been found in atherosclerotic plaques with new approaches to target periodontal bacteria's DNA, RNA and antigens (46). Furthermore, some scholars have found live *P. gingivalis* in atheroma or isolated *P. gingivalis* from cultured atheromatous tissue (5). To further demonstrate the role of periodontal infections in cardiovascular diseases, periodontal pathogens have been colonized on different animal models and proven to induce or promote atherosclerotic lesions. On the contrary, the noninvasive mutant of *P. gingivalis* did not promote atherosclerosis and induced fewer pro-inflammatory mediators than the wild type (47, 48).

Besides atherosclerosis, endocarditis is also believed to be associated with periodontitis (49). The bacteria present in the periodontal pockets can travel through the bloodstream and reach other parts of the body, including the heart (50, 51). The bacteria can form clumps or colonies on the valves, leading to the formation of infective endocarditis lesions. These lesions can cause inflammation and damage to the heart tissues. According to Herbert Deppe's study, 10% of bacterial sources of infective endocarditis are from oral pathogens (52). Furthermore, Rinaldo et al. also reported that infective endocarditis in patients with heart valve prostheses was mainly caused by oral bacteria in young male adults in Brazil and 77% of infective endocarditis cases were at high or moderate risk for periodontal disease (53).

Apart from the extensively studied roles, certain prominent subjects have significantly contributed to the interplay between periodontitis and CVD. However, those topics need further research to clarify their roles, such as COVID-19 and peri-implantitis.

In the aftermath of the COVID-19 pandemic, numerous studies (3) (54–56) have surfaced suggesting a potential connection between periodontitis and COVID-19, both of which appear to induce and exacerbate various cardiometabolic complications (57). The nexus between COVID-19 and PD is intricate and multifaceted, with evidence implying a shared inflammatory pathophysiology (58). This inflammation can instigate endothelial dysfunction, oxidative stress, and coagulation irregularities, thereby fostering the onset of cardiometabolic complications (59), including cardiovascular disease, type 2 diabetes, and metabolic syndrome. Several researchers have highlighted the possible role of oral bacteria and their byproducts in triggering systemic inflammation, thereby contributing to the progression of COVID-19 and associated cardiometabolic complications (35). Recent findings further underscore that individuals with periodontal disease (60) or cardiovascular disease (61) exhibit a higher susceptibility to severe COVID-19 infection. Therefore, in the current scenario of coexistence between humans and the COVID-19 virus, it is crucial to manage these underlying conditions to reduce the risk of severe outcomes in the event of a COVID-19 infection.

Peri-implantitis is characterized by inflammation, bleeding, bone loss, and potential implant failure which is deeply related to periodontal tissue. Some studies have indicated that there might be a correlation between the presence of peri-implantitis and an increased risk of cardiovascular disease (62, 63). It has been proposed that the chronic inflammation associated with peri-implantitis could contribute to the development or progression of cardiovascular conditions (64). Hom-Lay Wang's studies showed that 64.6% of peri-implantitis was found in CVD patients, while only 56.5% of peri-implantitis was found in the controls (65). Moreover, IL-1β and TNF-α were significantly higher in CVD patients, which were significant markers for peri-implantitis (66). Inflammation plays a crucial role in the pathogenesis of atherosclerosis, the underlying cause of many cardiovascular diseases. The theory suggests that the systemic inflammatory response triggered by peri-implantitis could potentially contribute to the development or worsening of cardiovascular problems.

While the exact nature of the relationship between periodontitis and cardiovascular disease is still being explored, research studies have shown an association between the two. It is important to note that association does not necessarily imply causation, and more research is needed to establish a definitive cause-and-effect relationship. Nevertheless, the current bibliometric studies above not only help us to understand the relationship between periodontitis and cardiovascular disease by analyzing the keyword trends in recent years, but also show us potential general directions for future research. Given that our study's primary recommendations encompass periodontal pathogens (notably *Porphyromonas gingivalis*), inflammation, and atherosclerosis, we propose that future research endeavors focus on these three trajectories. The exigency for research in these three dimensions becomes particularly evident in the concluding lines of our article.

This study provides a systematic summary of global publication trends, aiding scholars in identifying essential authors, institutions, and journals within the field of interest. Moreover, through keyword and co-citation clustering analysis, this study offers valuable guidance to researchers in selecting promising research directions within the subject area. Future research in this domain should aim to conduct longitudinal studies with larger sample sizes to provide more robust evidence of the relationship between periodontitis and CVD. Rigorous clinical trials will help determine whether treating periodontal disease has a tangible impact on cardiovascular health and vice versa. Positive findings from such trials could revolutionize treatment approaches, leading to integrated management plans that address both diseases simultaneously. This holistic approach has the potential to yield improved patient outcomes and quality of life. Additionally, fostering multidisciplinary collaboration among dental practitioners, cardiovascular specialists, immunologists, and researchers from related fields can foster a robust exchange of knowledge and innovative ideas. The collective expertise and resources can accelerate the pace of research, driving forward novel discoveries and therapeutic breakthroughs. Anticipated future collaborations among authors, institutions, and countries are likely to expedite the development of research in the realm of periodontitis and CVD.

## 5. Limitation

The study is not without limitations. First, due to constraints in the currently utilized scientometric software, combining multiple databases for analysis proves challenging. Consequently, this study relied solely on the WoSCC database for literature screening, potentially leading to the omission of relevant publications. To address this limitation, future efforts will involve the inclusion of additional databases for analysis. Second, the inclusion of only English articles in this article resulted in a reduced pool of retrieved articles. Third, certain recently published literature updates were regrettably omitted. Furthermore, the lack of a unified standard for configuring CiteSpace, VOSviewer, and Bibliometrix settings introduces the possibility of deviations in the visual analysis. Additionally, the uneven quality of the collected literature data can compromise the credibility of the generated atlas.

## 6. Conclusion

In conclusion, the results of the visual analysis provide a comprehensive overview of the current landscape of research on periodontitis and CVD, offering valuable insights for future research directions. The highest number of articles originated from the USA, Japan, and Sweden; the University of Helsinki and Karolinska Institute emerged as the leading publishers; and eminent scholars include professors D'Aiuto, Francesco and Beck, James D. Current research targets the periodontitis-CVD mechanism, highlighting microbial co-initiators like *Porphyromonas gingivalis* and the key shared mechanism of inflammation. The strong association between atherosclerosis and periodontitis is well-established. However, it requires more research for a definitive cause-and-effect relationship between PD and CVD. Furthermore, it is crucial for future research to explore the development of therapeutic management strategies for both two diseases that effectively balance systemic and oral health care. Additionally, fostering collaborative relationships among authors and regions to establish standardized industry-recognized guidelines and consensus are highly necessary.

## Data Availability

The raw data supporting the conclusions of this article will be made available by the authors, without undue reservation.
