# Supplementary material for: Bibliometric research on analysis of links between periodontitis and cardiovascular diseases
Source: Front Cardiovasc Med. 2023 Sep 4;10:1255722. doi: 10.3389/fcvm.2023.1255722 (PMC10512184; doi:10.3389/fcvm.2023.1255722)
Supplement: Supplementary file 1 [file Table1.docx]

Supplementary Material

Bibliometric Research on Analysis of Links between Periodontitis and Cardiovascular Diseases

Kuangyun Tang^1, †^, Yongjia Wu^1, †^, Qianhan Zheng^1^, Xuepeng Chen^1,*^

^1^ Stomatology Hospital, School of Stomatology, Zhejiang University School of Medicine, Clinical Research Center for Oral Diseases of Zhejiang Province, Key Laboratory of Oral Biomedical Research of Zhejiang Province, Cancer Center of Zhejiang University, Hangzhou, Zhejiang, 310006, China

*** Correspondence:**Xuepeng Chen

Email: cxp1979@zju.edu.cn

†These authors contributed equally to this work

# Supplementary Figures


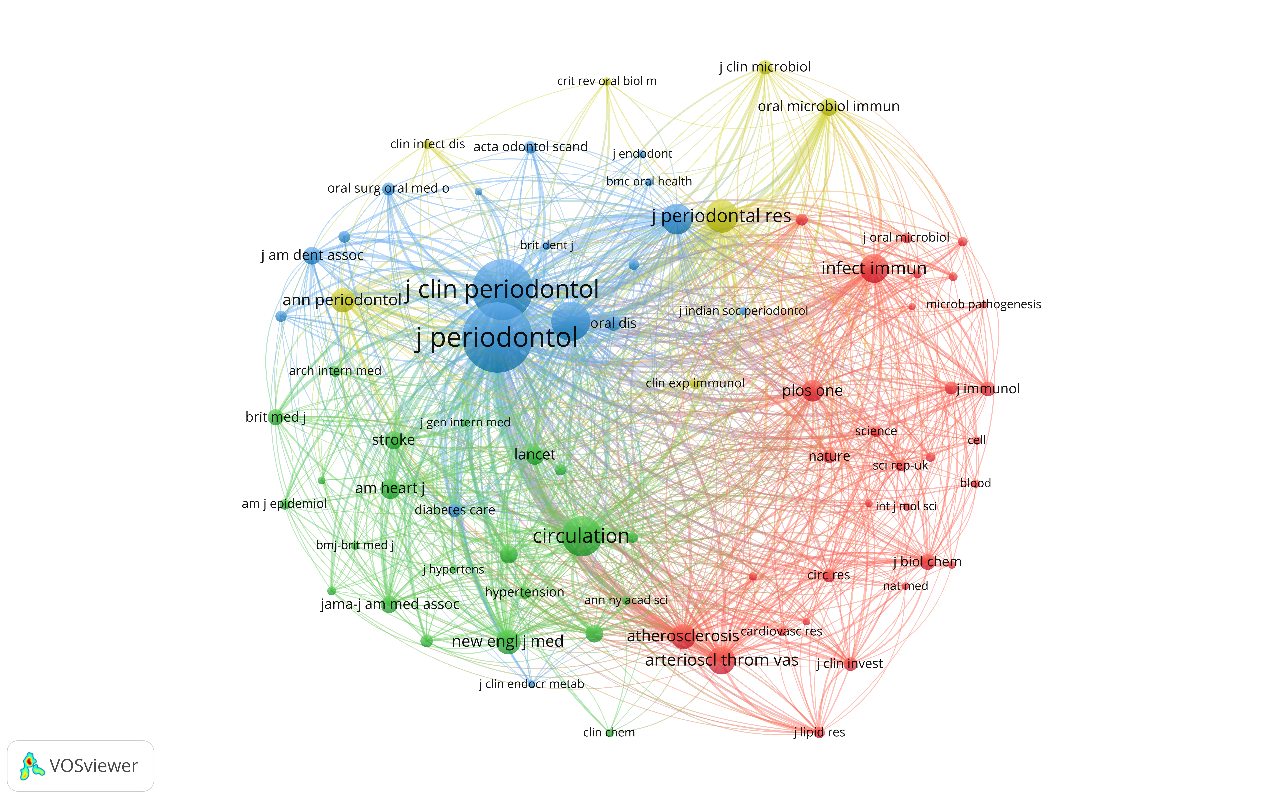


**Supplementary Figure S1.** Network visualization diagram of the journal co-citation analysis created by the VOS viewer.


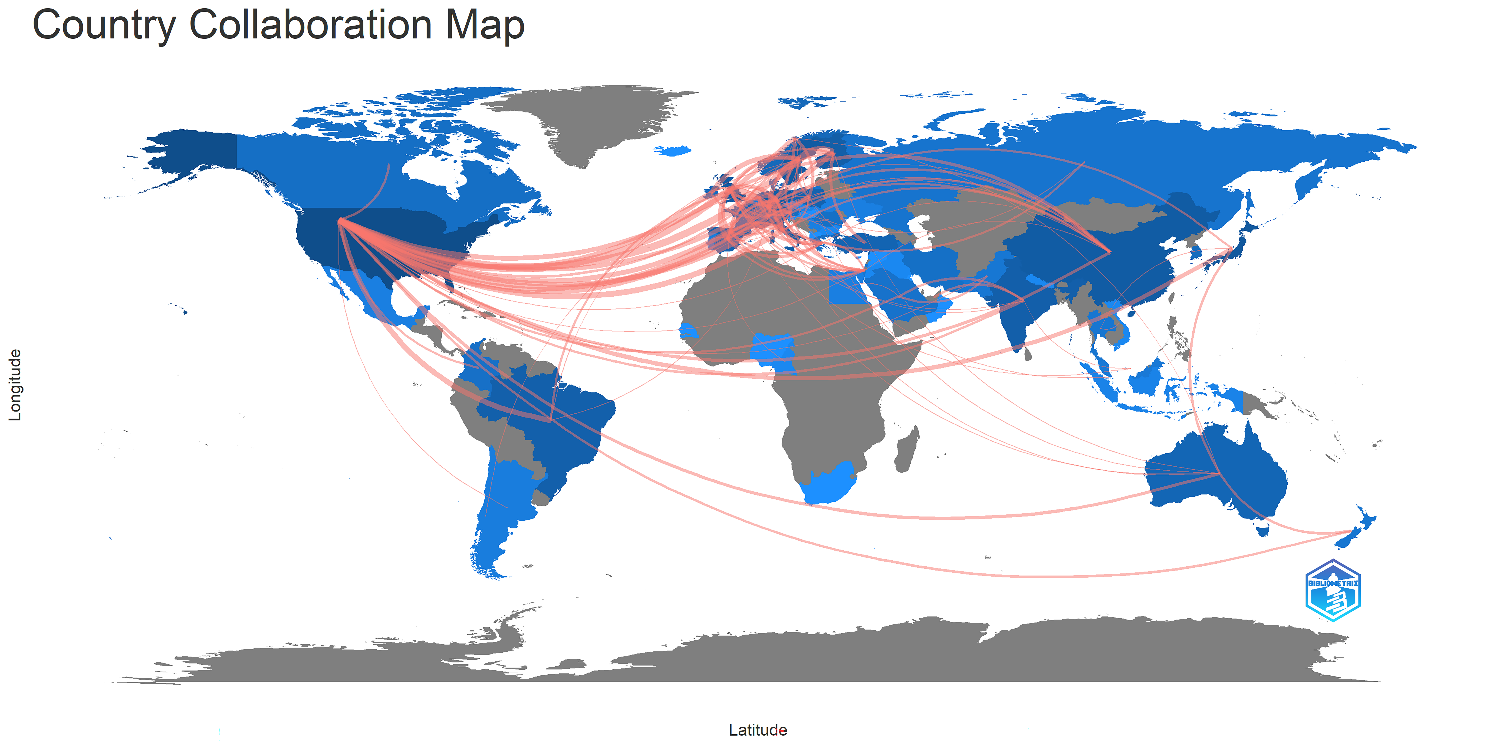


**Supplementary Figure S2.** The global distribution map of countries generated using R-Bibliometrix

**Supplementary S3**. PD-related terms. Periodontitis, Periodontal, Gingival, Peri-Implantitis, Periodontal Pockets, Tooth Loss, Tooth Migration, Tooth Drifting, Mesial Movement of Teeth, Tooth Mobility, Alveolar Bone Loss, Alveolar Resorption.

**Supplementary S4**. CVD-related terms. Cardiovascular, Atherosclerosis , Atherosclerotic, Angina, Acute Coronary Syndrome, Coronary Disease, Cardiac Arrhythmia, Carcinoid Heart Disease, Cardiac Conduction System Disease, Cardiac Tamponade, Cardiomegaly, Cardiomyopathies, Cardiotoxicity, Endocarditis, Heart Aneurysm, Heart Arrest, Heart Abnormality, Heart Failure, Heart Neoplasms, Heart Rupture, Heart Valve Diseases, Myocardial Ischemia, Myocardial Stunning, Myocardial infarction, Pericardial Effusion, Pericarditis, Pneumopericardium, Post-Cardiac Arrest Syndrome, Postpericardiotomy Syndrome, Pulmonary Heart Disease, Rheumatic Heart Disease, Ventricular Dysfunction, Ventricular Outflow Obstruction.
